# Psychological Features in the Inflammatory Bowel Disease–Irritable Bowel Syndrome Overlap: Developing a Preliminary Understanding of Cognitive and Behavioral Factors

**DOI:** 10.1093/crocol/otab061

**Published:** 2021-08-26

**Authors:** Megan Petrik, Brooke Palmer, Alexander Khoruts, Byron Vaughn

**Affiliations:** 1 University of Minnesota Medical School, Department of Medicine, Division of General Internal Medicine, Minneapolis, Minnesota, USA; 2 University of Minnesota Medical School, Department of Medicine, Division of Gastroenterology, Hepatology, and Nutrition, Minneapolis, Minnesota, USA

**Keywords:** inflammatory bowel disease, irritable bowel syndrome, chronic illness, psychogastroenterology

## Abstract

**Background:**

Inflammatory bowel disease (IBD) patients in clinical remission may experience ongoing symptoms, such as diarrhea and abdominal pain, attributed to IBD–irritable bowel syndrome (IBS) overlap. This study aims to characterize the psychosocial needs of patients with IBD–IBS overlap, particularly in regard to cognitive and behavioral functioning.

**Methods:**

Adults with an established IBD diagnosis were recruited from a gastroenterology clinic. Participants completed self-report questionnaires about psychological functioning and quality of life. The Rome IV Diagnostic Questionnaire for Adults-IBS Module assessed IBS criteria. The treating gastroenterologist completed a clinician rating of IBD activity to determine clinical disease activity. Biomarkers of inflammation collected in routine care within 90 days of the research encounter were obtained via medical record review to further contextualize IBD activity status. Participants were separated into the following groups: “inactive IBD” (IBD activity rating indicating inactive disease and no IBS criteria met), “active IBD” (IBD activity rating indicating mild, moderate, or severe regardless of IBS criteria), or “IBD–IBS overlap” (IBD activity rating indicating inactive disease and IBS criteria met).

**Results:**

One hundred and seventeen participants were recruited. Those with IBD–IBS overlap reported no significant differences in ratings of anxiety, depression, somatization, catastrophic thinking patterns, and behavioral avoidance, to patients with active IBD whereas participants with inactive IBD reported significantly lower ratings on these factors. However, a significant portion of participants with IBD–IBS overlap who were rated as inactive on IBD activity measures had laboratory or endoscopic findings indicating mild inflammation within 90 days of the research encounter.

**Conclusions:**

The study findings provide preliminary evidence that suggests patients with IBD–IBS overlap display similar rates of psychological distress, catastrophic thinking, and avoidance behaviors as those with active IBD. Those with mild ongoing inflammation despite meeting a definition for clinical remission may have similar psychological needs compared to those with moderate-to-severely active IBD. Incorporating a mental health provider with training in psychogastroenterology can help a patient with IBD learn how to effectively with these cognitive and behavioral patterns.

## Introduction

Inflammatory bowel diseases (IBD) are chronic digestive conditions associated with significant disease burden and healthcare costs.^1^ An emerging area of concern is understanding the prevalence and impact of the co-occurrence between IBD and irritable bowel syndrome (IBS). Approximately 3.1 million American adults have been diagnosed with an IBD.^2^ Between 20% and 44% of patients with controlled IBD experience ongoing gastrointestinal (GI) symptoms such as diarrhea and abdominal pain that are consistent with IBS.^3,4^ IBS is a disorder of gut–brain interaction that can persist in IBD patients despite apparent clinical disease control. Symptoms in IBS arise from complex and reciprocal interactions between biological, psychological, and social factors such as alterations in gut signaling that lead to normal physiologic stimuli being interpreted as painful (visceral hypersensitivity), cognitive-affective processes (eg, catastrophic thinking in regard to GI symptoms), and avoidance of stimuli that provoke fear of GI symptoms.^5,6^ IBD–IBS overlap is associated with anxiety, depression, fatigue, sleep disturbance, pain interference, narcotic use, and increased healthcare utilization even when IBD is in remission,^4^ necessitating management beyond suppression of inflammation.

When evaluating ongoing GI symptoms in IBD patients, it is essential to determine if symptoms are driven by inflammation or by gut–brain dysfunction, as treatment needs to be tailored to the appropriate pathophysiological mechanism. Gastroenterologists may erroneously escalate medical therapy for IBD when symptoms may be caused by IBS. This results in decreased cost-effectiveness of care and potential additional risks of IBD medications (eg, infections, malignancy)^7^ without benefits. Determining effective therapies for the subpopulation of IBD patients with IBS may improve patients’ GI symptoms and quality of life as well as reduce healthcare utilization and opioid misuse. It is common practice to use clinical disease index measures to estimate IBD activity, yet these clinical indices can overlap with IBS symptoms.^8^ Therefore, when disease activity indices are high, providers often look to objective measure of inflammation. However, when disease activity indices are low, providers will often assume residual or atypical GI symptoms are related to IBS or other functional bowel disease.

Interventions that target the gut–brain axis are likely to be the most effective for patients presenting with an IBD–IBS overlap. Cognitive behavioral therapy (CBT) is an effective psychological treatment for IBS, with improvement in GI symptoms from CBT often exceeding those of pharmacological interventions.^9^ While there is a robust literature documenting the efficacy of behavioral interventions and the impact of psychosocial factors on IBS,^6^ less is known about the psychological needs of patients with IBD–IBS overlap. Previous research in the United Kingdom found that IBS in IBD patients without objective markers of inflammation negatively impacted anxiety, depression, somatization, and quality of life to the same degree as active IBD.^10^ However, little is known about how cognitive and behavioral factors influence the IBD–IBS overlap.

Catastrophic thinking (eg, overestimating the likelihood of a negative outcome while underestimating one’s ability to cope if a problem did occur) is an important cognitive factor influencing IBS as it predicts quality of life above and beyond IBS symptom severity or psychological well-being.^11^ Patients with IBS have higher rates of catastrophic thinking patterns compared to those with Crohn’s disease (CD) despite similar rates of GI symptom severity and visceral sensitivity.^12^ Behavioral patterns, such as avoidance of situations that evoke GI-specific anxiety, influence the maintenance of IBS.^13^ However, no research has examined cognitive and behavioral coping styles in the IBD–IBS overlap. In order to improve the clinical outcomes of patients with IBD–IBS overlap, it is necessary to understand the impact of cognitive and behavioral factors that may influence disease experience.

This study aimed to characterize the psychosocial needs of patients with IBD–IBS overlap by examining emotional, cognitive, and behavioral functioning as well as quality of life. Rates of anxiety, depression, somatization, quality of life, catastrophic thinking patterns about GI symptoms, and behavioral avoidance of stimuli that evoke GI-specific fears in participants with IBD–IBS overlap were obtained and compared to participants with active IBD and inactive IBD. Based on past research,^10^ it is anticipated that participants with IBD–IBS overlap will have similar ratings of anxiety, depression, somatization, and quality of life to those with active IBD. As the literature has identified catastrophic thinking patterns and behavioral avoidance as common features of IBS,^14^ it is anticipated these patterns may arise in the IBD–IBS overlap. However, these cognitive and behavioral patterns have not been evaluated in the context of IBS when it co-occurs with IBD. Further understanding catastrophizing and behavioral avoidance may inform psychological treatments for patients with IBD–IBS overlap as these factors are known to contribute to gut–brain dysfunction and are main targets of treatment in CBT for IBS.^14,15^

## Materials and Methods

### Participants and Setting

Participants were included if they were adults (ages 18 and over) with an established radiologic, histologic, or endoscopic diagnosis of IBD (CD or ulcerative colitis [UC]) as determined by their treating provider, who received treatment through the University of Minnesota IBD Program. Exclusion criteria included the following: inability to complete questionnaires in the English language and patients who previously designated themselves as not to be approached about research conducted within the healthcare system. Those with an end ileostomy, colostomy, subtotal colectomy, or ileal pouch anal anastomosis were excluded as the clinical disease activity indices are not accurate in these subpopulations.

### Procedure

Participants were consecutively screened for inclusion and exclusion criteria and approached for participation by a member of their treatment team if criteria were met. If they agreed to participate, informed consent was obtained. Participants completed self-report questionnaires that assessed psychological factors and quality of life. Information about patient demographics as well as current alcohol and tobacco use was obtained via self-report. The treating gastroenterology provider specializing in IBD completed a clinician rating of IBD disease activity. Medical record review was used to obtain relevant IBD information. Completion of the self-report questionnaires took approximately 25 minutes and participants received a $25 gift card for participating in the study. The study protocol was approved by the Institutional Review Board at the University of Minnesota.

### Measures of GI Functioning

#### IBD measures

IBD disease activity information was collected using noninvasive measures. IBD activity rating scales are traditionally utilized to help gastroenterologists determine treatment plans. These measures heavily rely on assessment of diarrhea and blood in the stool.

For patients with CD, the treating gastroenterology provider completed the Harvey–Bradshaw Index (HBI).^16^ The HBI measures patient well-being, abdominal pain, the number of liquid or soft stools, and the presence of abdominal mass and complications. HBI scores under 5 indicate inactive disease/remission, 5–7 indicate mild disease, 8–16 indicate moderate disease, and 17+ indicate severe disease.

The partial Mayo score^17^ assessed disease activity for patients with UC. Patients rated their stool frequency and rectal bleeding, while the treating gastroenterology provider completed the global assessment of disease. This is a shortened version of the Mayo score, which has been used in many clinical trials for UC, that omits consideration of endoscopic findings. The partial Mayo score has been shown to accurately predict the Mayo score,^18^ suggesting the ability to measure disease activity without endoscopy. Scores of 0–1 indicate inactive disease/remission, 2–4 indicate mild disease, 5–6 indicate moderate disease, and 7–9 indicate severe disease.

While the study was designed to use clinician ratings as the primary measure of disease activity, medical record review was also performed to gather relevant objective measures of inflammation to provide additional context to the findings. This included gathering information about imaging, endoscopy, serum biomarkers of inflammation, and fecal biomarkers of inflammation obtained within ±90 days of the research encounter.

#### IBS measures

The Rome IV Diagnostic Questionnaire for Adults-IBS Module^19^ is a 9-item self-report questionnaire that assessed diagnostic criteria for IBS. This measure has adequate sensitivity and excellent specificity for diagnosis of IBS and has good test–retest reliability. When IBS criteria was met, the Irritable Bowel Syndrome-Severity Scoring System (IBS-SSS^20^), a 5-item validated measure of IBS symptom severity,^21^ was also completed. Participants rated the intensity of abdominal pain, distension, stool frequency and consistency, and interference with life in the last 10 days on a 0–100 visual analog scale. Scores were obtained by summing responses to the items, with a maximum possible score of 500. Scores between 75 and 175 indicate mild IBS, 175 and 300 indicate moderate IBS, and over 300 indicates severe IBS.^20^

#### IBD–IBS overlap categorization

Participants were separated into the following groups based on their scores on IBD and IBS measures: (1) *IBD–IBS overlap*: clinician rating of IBD activity indicating inactive IBD, while IBS criteria was met on participant self-report on Rome IV Diagnostic Questionnaire; (2) *inactive IBD*: clinician rating of IBD activity rating indicating inactive disease and IBS criteria not met on participant self-report on Rome IV Diagnostic Questionnaire; and (3) *active IBD*: clinician rating of IBD activity rating indicating mild, moderate, or severe regardless of IBS criteria. To have been categorized in the IBD–IBS overlap group, participants’ IBD clinical disease activity rating needed to be inactive. IBS was only recognized if participants’ clinician rating of disease activity was inactive.

### Measures of Psychosocial Functioning

#### Short-Form Health Survey

The Short-Form Health Survey (SF-36) is a validated 36-item self-report measure that assessed health-related quality of life.^22–24^ Reliability for the SF-36 has been demonstrated to be good across a variety of patient populations (median reliability coefficients = .85).^23^ In this study, internal consistency of the SF-36 was strong (Cronbach’s α = .95). Each item is scored on a 0–100 range, with higher scores indicating more optimal quality of life. The SF-36 includes the following 8 scales: physical functioning, role limitations due to physical health problems, role limitations due to emotional problems, emotional well-being, bodily pain, social functioning, energy/fatigue, and general health problems. The SF-36 has also been used in previous studies examining psychological health and quality of life in the IBD–IBS overlap^10,25^ and has been found to be valid in both IBD^26^ and IBS^27^ patient populations. The SF-36 was selected to assess quality of life for patients with both IBD and IBS diagnoses.

#### Hospital Anxiety and Depression scale

Hospital Anxiety and Depression scale (HADS) is a 14-item questionnaire that measured the presence of anxiety and depressive symptoms with 7 items for each domain.^28^ This measure was designed to assess anxiety and depression in medical populations by removing somatic complaints and has been used in studies evaluating co-occurring IBD and IBS.^10,25^ Each item was rated on a 4-point Likert scale. Scores for both anxiety and depression range from 0 to 21. Scores of 0–7 fall within the normal range, 8–10 indicate borderline normal symptoms, and scores ≥11 are in the abnormal range. A review of the validity of the HADS across a variety of patient populations suggested good internal consistency for both the anxiety (mean Cronbach’s α = .83) and depression (mean Cronbach’s α = .82) subscales.^29^ In this study, internal consistency of the anxiety (Cronbach’s α = .85) and depression (Cronbach’s α = .83) subscales was good.

#### Patient Health Questionnaire-15

Somatization was assessed using the Patient Health Questionnaire-15 (PHQ-15), in which participants rated the severity of 15 somatic symptoms over the last 4 weeks on a 0–2 Likert scale (0 = “not bothered at all”, 2 = “bothered a lot”).^30^ Scores of less than 4 indicate minimal somatization, 5–9 = low, 10–14 = medium, and 15+ indicate high. This scale comes from the validated full Patient Health Questionnaire^31^ and the symptoms have been shown to account for the over 90% of the physical complaints reported in outpatient healthcare encounters.^32^ Internal consistency for the PHQ-15 has been found to be good (Cronbach’s α = .80).^30^ In this study, internal consistency of the PHQ-15 was adequate (Cronbach’s α = .76).

#### GI-Cognitions Questionnaire

GI-Cognitions Questionnaire (GI-Cog) is a 16-item self-report questionnaire that was used to measure catastrophic thoughts about the implications of GI symptoms as they pertain to embarrassment in social settings, perceived incompetence, avoidance and interference with activities, and urgency/incontinence.^12^ Participants were asked to rate items on a 5-point Likert scale ranging from 0 (hardly at all) to 4 (very much). Example items include “If I feel the urge to defecate and cannot find a bathroom right away I won’t be able to hold it and I’ll be incontinent” and “If people knew about my gut problems, they would think about me negatively.” The scale is scored by summing all responses (range = 0–64). Scores between 0 and 19 indicate mild catastrophizing, 20 and 39 = moderate, 40 and 64 = severe. The GI-Cog has been shown to have excellent internal consistency (Cronbach’s α = .92) and good 1 week test–retest reliability (*r* = .87), and has been used with both IBS and IBD populations.^12^ Internal consistency of the GI-Cog in this study was good (Cronbach’s α = .88).

#### Irritable Bowel Syndrome-Behavioral Responses Questionnaire

The Irritable Bowel Syndrome-Behavioral Responses Questionnaire (IBS-BRQ) is a 28-item questionnaire that measures behaviors related to GI symptoms that are common in IBS, such as avoidance and specific toileting behaviors.^33^ Participants were asked to rate items on a 7-point Likert scale that indicated how often the behavior occurs (1 = never, 7 = always). Example items include “I avoid making plans in case I have problems with my IBS” and “I am constantly aware of my stomach.” This measure was adapted to replace the term IBS with “GI functioning” to allow comparisons of avoidance behaviors between participants with IBD–IBS overlap and those with only IBD. The scale is scored by summing all responses (range = 29–203). Internal consistency reliability for the IBS-BRQ has been shown to be good for both IBS (Cronbach’s α = .86) and control groups (Cronbach’s α = .89).^33^ Additional validation of the IBS-BRQ shows strong test–retest reliability in IBS patients (*r* = .81–.85) in 3-, 6-, and 12-month follow-up periods as well as good criterion and discriminant validity.^33^ Internal consistency of the IBS-BRQ in this study was strong (Cronbach’s α = .92).

### Statistical Analysis

First, differences between participants with CD and UC were evaluated on demographic and medical characteristics, psychological functioning, and quality of life. Independent samples *t*-tests were used for continuous variables and the chi-square test of independence was used for categorical variables. Next, differences between participants with IBD–IBS overlap, inactive IBD, and active IBD were evaluated on demographic and medical characteristics, psychological functioning, and quality of life. One-way ANOVAs were used for continuous variables and the chi-square test of independence was used for categorical variables. Statistical significance for tests examining psychosocial variables as well as demographic/medical factors was met using 2-tailed tests and a *P* value of <.003 due to use of multiple comparisons via Bonferroni correction.^34^ An assumption of the chi-square test is that at least 80% of the cells will have an expected value of 5 or more and when this assumption was violated a 2-sided Fisher’s exact test was conducted for analyses of 2 × 2 tables and a maximum likelihood ratio chi-square test was conducted for crosstabulations larger than 2 × 2.^35^ Correlation analyses were also conducted to examine variables of interest in light of a small sample size for the IBD–IBS overlap group. All analyses were conducted using SPSS for Mac, Version 27. An a priori power analysis was conducted using G*Power. For analyses comparing mean scores of psychological variables (eg, anxiety, depression, somatization, and quality of life) assuming a large effect size^10^ (*f* = 0.4) and using a 1-way ANOVA with 3 groups and a Bonferroni corrected significance level of 0.003, a total sample size of 114 would allow for 80% power.

## Results

### Participant Characteristics

One hundred and twenty individuals consented to participate. Three participants were excluded due to current ostomy (*n* = 2) and no IBD diagnosis upon later clinician review (*n* = 1), leaving 117 participants for analysis. Of the 117 participants, 58% had a diagnosis of CD (*n* = 68) and 42% had a diagnosis of UC (*n* = 49). There were no significant differences between participants with CD versus UC on age, gender, race, ethnicity, relationship status, employment status, alcohol and tobacco use, length of time with IBD diagnosis, and percentage with IBS. Approximately 19% of participants with CD and 10% of those with UC met criteria for IBS based on self-report on the Rome IV Diagnostic Questionnaire for Adults-IBS Module. However, IBS diagnostic criteria were not considered to be met for patients with active IBD status as active disease of any severity precluded the ability to accurately diagnose IBS. CD participants were more likely to have had a history of a surgical resection. There were differences between participants with CD and UC in medication use; participants with CD were more likely to be using biologic medications whereas participants with UC were more likely to be currently using aminosalicylates, which reflects current practices in management based on disease phenotype. There were no significant differences between participants with CD and UC regarding symptoms of anxiety and depression, somatization, quality of life, catastrophic thinking patterns, or behavioral responses. Supplementary Table 1 displays comparisons of demographic/medical characteristics by IBD diagnosis whereas Supplementary Table 2 displays comparisons of psychological functioning and quality of life by IBD diagnosis.

### Comparisons of Characteristics in IBD Participants With and Without IBD–IBS Overlap

#### Demographic and medical characteristics

Table 1 displays comparisons of demographic and medical characteristics in IBD participants with and without the IBD–IBS overlap. There was no association on gender, race, ethnicity, relationship status, employment status, current use of alcohol or tobacco products, history of surgical resection, and current medications used between participants with an IBD–IBS overlap, inactive IBD, and active IBD. There were also no significant differences between these groups on age and length of time with IBD diagnosis.

**Table 1.** Comparisons of demographics and medical characteristics in participants with and without IBD–IBS overlap

	IBD–IBS overlap	Inactive IBD	Active IBD	Statistical test
*n*	8	69	33	
Age (yrs), *M* (SD)	37.8 (15.9)	38.9 (16.5)	36.1 (11.1)	*F*(2, 109) = .42, *P* = .66
Gender, *n* (%)				χ ^2^(2, 110) = 3.34, *P* = .24*
Male	1 (12.5)	30 (43.5)	14 (42.4)	
Female	7 (87.5)	39 (56.5)	19 (57.6)	
Race, *n* (%)				χ ^2^(6, 110) =7.93, *P* = .22*
Asian	0 (0.0)	0 (0.0)	1 (3.0)	
Black	0 (0.0)	1 (1.4)	2 (6.1)	
White	7 (87.5)	65 (94.2)	30 (90.6)	
Biracial	1 (12.5)	3 (4.3)	0 (0.0)	
Hispanic ethnicity, *n* (%)	1 (12.5)	3 (4.4)	0 (0.0)	χ ^2^(2, 109) = 3.67, *P* = .33*
Relationship status, *n* (%)				χ ^2^(4, 110) = 2.42, *P* = .70*
Single	3 (37.5)	25 (36.2)	8 (24.2)	
Married or committed relationship	5 (62.5)	41 (59.4)	24 (72.7)	
Divorced/widowed	0 (0.0)	3 (4.3)	1 (3.0)	
Employed full or part time, *n* (%)	7 (87.5)	55 (79.7)	21 (63.6)	χ ^2^(2, 110) = 3.79, *P* = .15
Current alcohol use, *n* (%)	4 (50.0)	54 (78.3)	21 (63.6)	χ ^2^(2, 110) = 4.39, *P* = .11
Current tobacco use, *n* (%)	1 (12.5)	4 (5.8)	3 (9.1)	χ ^2^(2, 110) = .66, *P* = .88*
Years with IBD diagnosis, *M* (SD)	13.1 (16.5)	10.5 (10.0)	9.45 (7.9)	*F*(2, 109) = .44, *P* = .64
History of surgical resection, *n* (%)	3 (37.5)	11 (15.9)	12 (36.4)	χ ^2^(2, 110) = 6.08, *P* = .05
Current use of aminosalicylate therapy, *n* (%)	2 (25)	24 (34.8)	8 (24.2)	χ ^2^(2, 110) = 1.30, *P* = .52
Current use of corticosteroid therapy, *n* (%)	0 (0.0)	6 (8.7)	7 (21.2)	χ ^2^(2, 110) = 5.04, *P* = .08*
Current use of immunomodulator therapy, *n* (%)	3 (37.5)	17 (24.6)	7 (21.2)	χ ^2^(2, 110) = .92, *P* = .63
Current use of biologic therapy, *n* (%)	5 (52.5)	38 (55.1)	16 (48.5)	χ ^2^(2, 110) = .66, *P* = .75*
IBS-Symptom Severity Score (SSS), *M* (SD)	217.12 (82.6)			
IBS-SSS categories, *n* (%)				
Mild	2 (25.0)			
Moderate	4 (50.0)			
Severe	2 (25.0)			

Statistical significance was met using a *P* value of <.003 due to Bonferroni correction to adjust for multiple comparisons. Abbreviation: IBD–IBS, inflammatory bowel disease–irritable bowel syndrome.

*Maximum likelihood ratio chi-square test.

On further physician chart review, 6 of the 8 participants with IBD–IBS overlap had evidence of mild abnormalities on laboratory testing (as defined by fecal calprotectin <100 µg g^−1,36^ erythrocyte sedimentation rate <8 mg L^−1^, C-reactive protein <2.9 mm hr^−1^), radiologic imaging (computed tomography or magnetic resonance enterography), or endoscopic evaluation within 90 days of this research encounter. These 6 participants were not reclassified based on medical record review in order to keep the initial design of the study intact (eg, to measure clinical disease activity systematically using clinician ratings at the time of the research encounter). Yet, we included information about the most recently collected biomarkers of inflammation to provide additional context for results. The aim in doing this is to inform results for gastroenterologists treating patients with an IBD–IBS overlap.

#### Psychological functioning

**Figure 1.**
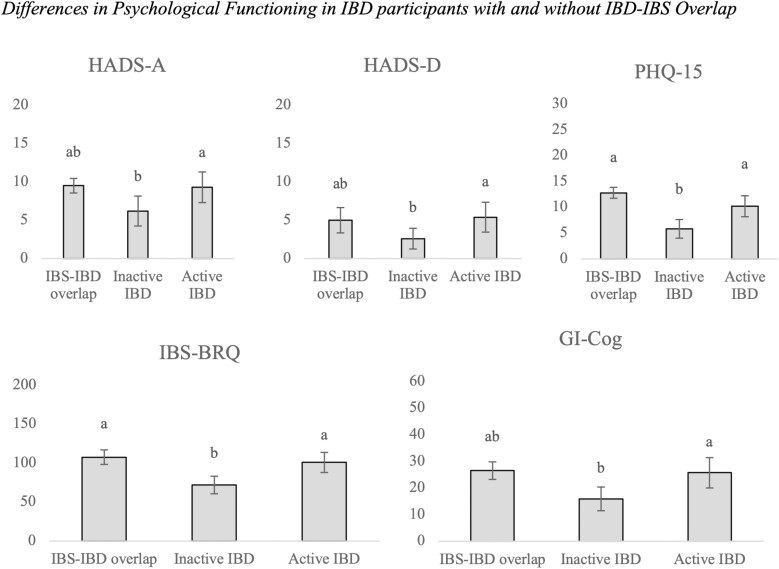
Data presented are mean differences (SD) in psychological functioning in IBD participants with and without IBD–IBS overlap. Different letters denote significant differences between groups. Abbreviations: GI-Cog, GI-Cognitions Questionnaire; HADS-A, Hospital Anxiety and Depression scale-anxiety subscale; HADS-D, Hospital Anxiety and Depression scale-depression subscale; IBD–IBS, inflammatory bowel disease–irritable bowel syndrome; IBS-BRQ, Irritable Bowel Syndrome-Behavioral Responses Questionnaire; PHQ-15, Patient Health Questionnaire-15. Groups that share the same letter indicate no statistically significant differences between groups whereas groups that have different letters indicate statistically significant differences between groups.

There were significant differences between participants with IBD–IBS overlap, inactive IBD, and active IBD for symptoms of anxiety (*η*^2^ = .13), depressive symptoms (*η*^2^ = .14), somatization (*η*^2^ = .31), catastrophic thinking patterns (*η*^2^ = .22), and behavioral avoidance patterns (*η*^2^ = .29; see Table 2). LSD post hoc tests revealed that participants with IBD–IBS overlap reported no significant differences in ratings of anxiety, depression, somatization, catastrophic thinking patterns, and behavioral avoidance to patients with active IBD (*P*s range between .06 and .83). Participants with inactive IBD reported significantly lower ratings than those with active IBD on all of these factors (*P*s < .001). Participants with inactive IBD also reported lower ratings than those with the IBD–IBS overlap on somatization and behavioral avoidance (*P*s < .001); no significant differences between these groups were found on anxiety (*P* = .02), depression (*P* = .04), and catastrophic thinking patterns (*P* = .003). Figure 1 displays these differences.

**Table 2.** Comparisons of psychological functioning and quality of life in participants with and without IBD–IBS overlap

	IBD–IBS overlap	Inactive IBD	Active IBD	Statistical test
*n*	8	69	33	
HADS anxiety score, *M* (SD)	9.5 (1.9)	6.2 (3.9)	9.3 (4.0)	*F*(2, 107) = 8.93, *P* < .001
HADS depression score, *M* (SD)	5.0 (3.3)	2.6 (2.7)	5.4 (3.9)	*F*(2, 107) = 9.03, *P* < .001
PHQ-15 overall score, *M* (SD)	12.8 (2.12)	5.82 (3.61)	10.2 (4.06)	*F*(2, 108) = 24.13, *P* < .001
SF-36 scores, *M* (SD)				
Physical functioning	82.5 (22.3)	90.9 (16.8)	77.1 (25.9)	*F*(2, 109) = 5.29, *P* = .006
Role limits physical health	71.9 (41.2)	86.6 (29.6)	49.2 (43.1)	*F*(2, 109) = 12.54, *P* < .001
Role limitations emotional problems	66.7 (39.8)	77.9 (33.8)	55.2 (43.7)	*F*(2, 107) = 4.05, *P* = .02
Emotional well-being	67.0 (13.8)	75.2 (18.2)	60.2 (17.9)	*F*(2, 108) = 7.96, *P* < .001
Bodily pain	63.1 (17.6)	80.1 (19.3)	55.9 (29.9)	*F*(2, 108) = 12.95, *P* < .001
Social functioning	70.3 (14.8)	87.9 (20.2)	61.7 (25.9)	*F*(2, 109) = 16.66, *P* < .001
Energy/fatigue	50.6 (12.7)	58.5 (22.2)	37.7 (23.7)	*F*(2, 108) = 9.79, *P* < .001
General health problems	38.8 (21.3)	58.4 (18.7)	40.6 (21.6)	*F*(2, 109) = 10.85, *P* < .001
GI catastrophizing score, *M* (SD)	26.5 (6.6)	15.9 (8.9)	25.7 (11.4)	*F*(2, 109) = 13.88, *P* < .001
IBS-Behavioral Responses Questionnaire, *M* (SD)	107.6 (18.6)	71.9 (22.5)	100.8 (25.8)	*F*(2, 109) = 21.93, *P* < .001

Statistical significance was met using a *P* value of <.003 due to Bonferroni correction to adjust for multiple comparisons. Abbreviations: GI, gastrointestinal; HADS, Hospital Anxiety and Depression scale; IBD–IBS, inflammatory bowel disease–irritable bowel syndrome; PHQ-15, Patient Health Questionnaire-15; SF-36, Short-Form Health Survey.

#### Quality of life

**Figure 2.**
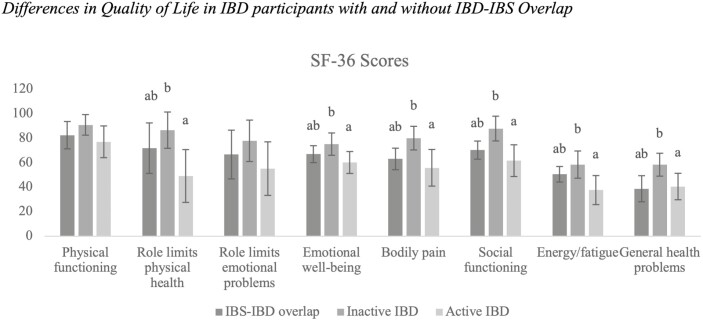
Data presented are mean differences (SD) in quality of life in IBD participants with and without IBD–IBS overlap. Different letters denote significant differences between groups. Abbreviations: IBD–IBS, inflammatory bowel disease–irritable bowel syndrome; SF-36, Short-Form Health Survey. Groups that share the same letter indicate no statistically significant differences between groups whereas groups that have different letters indicate statistically significant differences between groups.

There were significant differences between participants with an IBD–IBS overlap, inactive IBD, and active IBD for 6 of the 8 SF-36 scales: role limits on physical health (*η*^2^ = .19), pain (*η*^2^ = .19), social functioning (*η*^2^ = .22), energy/fatigue (*η*^2^ = .16), emotional well-being (*η*^2^ = .12), and general health (*η*^2^ = .16; see Table 2). Those with active IBD and the IBD–IBS overlap reported no significant differences on ratings of each of these SF-36 scales (*P*s range between .12 and .72). Those with active IBD rated each of these SF-36 as lower—indicating worse quality of life in these areas—than those with inactive IBD (*P*s < .001). There were no significant differences across these SF-36 scales between active IBD and those with IBD–IBS overlap (*P*s range between .009 and .34; see Figure 2).

### Additional Analyses Examining the Relationship Between Cognitive and Behavioral Factors and GI Functioning

Since the small size of the IBD–IBS overlap group impacted the interpretation of between group analyses, additional correlational analyses were conducted to examine the relationship between cognitive and behavioral factors and GI functioning. There were significant correlations between clinician ratings of IBD activity for those with CD and catastrophic thinking patterns (*r* = .42, *P* < .001) and behavioral avoidance (*r* = .47, *P* < .001). There also were significant correlations between clinician ratings of IBD activity for those with UC and catastrophic thinking patterns (*r* = .39, *P* = .008) and behavioral avoidance (*r* = .53, *P* < .001).

## Discussion

This paper provides an initial investigation into cognitive and behavioral factors in the IBD–IBS overlap as compared to IBD patients without IBS. Our study found that participants who met clinical criteria for remission from IBD yet met criteria for IBS had comparable anxiety, depression, somatization, and quality of life scores as those with clinically active IBD. On further review, a number of these individuals had evidence of mild inflammation. Treatment decisions for individuals classified as in clinical remission but with other indicators of mild inflammation are difficult. IBD therapy escalation is often performed for moderate-to-severe disease. Our study suggests that symptoms attributed to IBS have a psychological impact similar to active IBD and may be driven by mild ongoing inflammation.

Overall, our results were similar to Gracie et al’s^10^ findings in that ratings of anxiety, depression, somatization, and quality of life for those with IBD–IBS overlap were comparable to those with active IBD and generally worse that those with inactive IBD. We also found that maladaptive thinking patterns and behavioral avoidance in the IBD–IBS overlap were similar to patients with active IBD. Significant associations between cognitive and behavioral factors and IBD activity status were found, suggesting a need to further examine the relationship between these variables in future research. One discrepancy with past literature is the rate of co-occurring IBS in IBD in our study was lower than expected as past literature has found it to be between 20% and 44%.^3,4,10^ Rates for IBD–IBS for patients with CD in this study are comparable to other research that used self-report methodology to capture diagnosis.^4^ Additionally, rates of participants meeting diagnostic criteria for IBS in a global, large-scale study investigating prevalence of functional GI disorders were lower when using Rome IV criteria as compared to Rome III criteria.^37^ This is relevant as we used Rome IV criteria whereas past research by Gracie et al^10^ used Rome III criteria, which may in part account for this difference. Another difference between our study and past research was the method for measurement of active IBD. Gracie et al utilized disease activity ratings and fecal calprotectin to measure mucosal inflammation. Their main analyses utilized fecal calprotectin <250 mg g^−1^ to indicate remission from inflammation (normal range is <50 mg g^−1^). When fecal calprotectin levels of <100 mg g^−1^ were used to define no evidence of mucosal inflammation, results remained in the same direction but there were fewer statistical significances between groups. As our study suggests, this may indicate the importance considering the role of mild inflammation in treatment planning.

The finding that patients with IBD–IBS overlap have similar rates of catastrophic thinking patterns and behavioral avoidance to patients with active IBD has important implications for providers. First, it is essential to identify active inflammation in IBD patients and not rely completely on symptoms as a surrogate for inflammation. Patients with IBD–IBS overlap can have similarly poor quality of life to active IBD. It is unknown if mild ongoing inflammation is responsible for the IBS symptoms and impact on mental health. A multidisciplinary approach including mental health providers is needed address the patient holistically and determine if and when IBD or IBS therapy should be escalated.

Our findings suggest that catastrophic thinking patterns and behavioral avoidance may be seen in the IBD–IBS overlap. Incorporating a mental health provider with training in psychogastroenterology can help a patient with IBD learn how to effectively with these cognitive and behavioral patterns. Catastrophic thinking amplifies pain and GI symptoms, reduces pain inhibition, negatively impacts interpersonal relationships, and increases patient disability.^38–41^ Cognitive interventions from CBT can help a patient recognize the influence of distorted thinking patterns on their emotions and GI functioning and alter these maladaptive patterns based on evidence. Changes in catastrophizing have also been shown to partially mediate the efficacy of CBT for IBS.^42^ Avoidance behaviors are a major factor in perpetuating IBS symptoms^14^ and are best managed through CBT which incorporates gradual exposures and behavioral experiments to reduce avoidant coping. Outcome studies have found that exposure-based CBT interventions for IBS improves GI symptoms, quality of life, and pain catastrophizing.^43–45^

It is essential, however, for accurate identification of the contribution of inflammation to symptoms as certain patterns of coping may be adaptive in periods of active IBD, but no longer helpful once inflammation resolves. Applied in context of avoidance behaviors, preemptively searching for a bathroom and avoiding situations without easy access to one is adaptive when living with active IBD. However, when GI symptoms are due to IBD–IBS overlap, this avoidant coping strategy can perpetuate GI symptoms and distress via gut–brain dysregulation.^14^ A systematic review examining the relationship between coping styles and psychological well-being in patients with IBD found that use of problem-focused coping in IBD is associated with positive psychological outcomes whereas emotion-focused coping is associated with negative psychological outcomes.^46^ This finding is contrary to research supporting the notion that flexibly employing emotion-focused coping strategies in the context of IBS enhances outcomes.^47,48^

It is therefore important for treating providers to accurately identify patients with IBD–IBS overlap as the psychological management strategies vary from active IBD, despite similar mental health burdens. This highlights the importance of implementing flexible coping in responses to changes in disease status. As the symptoms of IBD and IBS can be indistinguishable, a patient would not likely know the etiology of their symptoms. It is essential to obtain guidance from their gastroenterologist about the reason for symptom presentations. Once this is known, treatments can be tailored to the appropriate pathophysiological mechanism. For the subset of patients with co-occurring IBD and IBS, there likely will need to be 2 different sets of strategies to enhance coping in each stage of disease as well as patient fluency in knowing when to use each strategy. It may be beneficial for patients with the IBD–IBS overlap to work with a mental health provider trained in psychogastroenterology to learn how to optimally cope with different disease states. In addition to improvement in quality of life, involving a GI mental health provider for patients with an IBD–IBS overlap would also likely reduce burden on medical providers who feel as if their medication-based interventions are not adequately managing symptoms. Future interventions examining the effectiveness of CBT in IBD–IBS overlap in reducing GI symptoms also would be of benefit.

Results should be taken into consideration in the context of several salient limitations to our study. First, disease activity may not accurately reflect true disease status as it was measured using clinician ratings rather than using systematically collected objective measures of inflammation. Therefore, patients classified in remission of IBD may have had ongoing mucosal inflammation that could perpetuate the gut–brain dysregulation. For 6 of the 8 participants in the IBD–IBS overlap category, clinical IBD activity ratings indicated remission, yet medical record review revealed testing suggestive of residual mild inflammation within 90 days of the research encounter. Disease activity ratings rely on assessment of diarrhea and blood in the stool and may not accurately capture mild inflammation. This speaks to the importance of future research analyzing the correlation between clinician ratings of IBD status and biomarkers of inflammation in the IBD–IBS population. It is possible that more aggressive treatment of inflammation detected by more sensitive tests could benefit symptoms that are attributed to IBS. Future research that simultaneously collects patient report of cognitive and behavioral factors and standardized assessments of mucosal inflammation is needed to tease this out.

Additionally, the size of the sample for patients with the IBD–IBS overlap was small and the generalization of findings is compromised. Nevertheless, effect sizes between the groups were large, suggesting there may be meaningful differences between IBD patients with and without IBS. The small sample size also led to violating the assumptions of chi-square tests and the use of alternative statistics. Violating the assumptions of statistical tests may lead to unreliable results, but it is of note that the chi-square tests of independence and alternative tests yielded the same results. Given the small size of the IBD–IBS overlap group, this study may be best utilized as a study that offers preliminary findings on cognitive and behavioral factors that impact the IBD–IBS overlap. Future research that addresses the methodologic limitations of our study is needed to replicate our findings. Another limitation was that certain measures (eg, Rome IV Diagnostic Questionnaire and the IBS-BRQ) have not been validated with IBD populations. Finally, our population was limited to English speaking patients only. It is unclear how generalizable our results are to other non-English speaking populations.

## Conclusions

This study provides a preliminary examination of how cognitive and behavioral factors such as catastrophic thinking and behavioral avoidance are present in the IBD–IBS overlap. Patients who present with co-occurring IBD and IBS appear to endorse anxiety, depression, somatization, catastrophic thinking, avoidance behaviors, and quality of life impairment to a similar degree as those with active IBD. Incorporating the assessment and treatment of these psychosocial factors may have a positive impact on GI outcomes and quality of life for patients with IBD–IBS overlap. Accurate identification of the contribution of inflammation to the symptom experience via biological testing is a key part of developing an effective treatment plan.

## Supplementary Material

otab061_suppl_Supplementary_Tables

## Data Availability

The data that support the findings of this study are available from the corresponding author, upon reasonable request.
