# Supplementary material for: Psychological Features in the Inflammatory Bowel Disease–Irritable Bowel Syndrome Overlap: Developing a Preliminary Understanding of Cognitive and Behavioral Factors
Source: Crohns Colitis 360. 2021 Aug 26;3(3):otab061. doi: 10.1093/crocol/otab061 (PMC9802046; doi:10.1093/crocol/otab061)
Supplement: otab061_suppl_Supplementary_Tables [file otab061_suppl_supplementary_tables.docx]

Supplemental Table 1. Participant demographics and medical characteristics by type of IBD diagnosis.

|  | Crohn’s Disease | Ulcerative Colitis | Statistical test |
| --- | --- | --- | --- |
| *n* | 68 | 49 |  |
| Age (yrs), M (SD) | 38.0 (15.9) | 39.1 (14.2) | *t*(115) = -.38, *p* = .70 |
| Gender, n (%) |  |  | χ^2^(1, 117) = .52, *p* = .47 |
| Male | 26 (38.2) | 22 (44.9) |  |
| Female | 42 (61.8) | 27 (55.1) |  |
| Race, n (%) |  |  | χ^2^(4, 117) = 3.43, *p* = .48* |
| Asian | 0 (0.0) | 1 (2.0) |  |
| Black | 3 (4.4) | 1 (2.0) |  |
| White | 62 (91.2) | 45 (91.8) |  |
| Biracial | 2 (2.9) | 2 (4.1) |  |
| Hispanic ethnicity, n (%) | 4 (6.0) | 0 (0.0) | *p* = .14^ |
| Relationship status, n (%) |  |  | χ^2^(2, 117) = 1.08, *p* = .65 |
| Single | 24 (35.3) | 13 (26.5) |  |
| Married or committed relationship | 41 (60.3) | 34 (69.4) |  |
| Divorced/widowed | 3 9(4.4) | 2 (4.1) |  |
| Employed full or part time, n (%) | 49 (72.1) | 38 (77.6) | χ^2^(1, 117) = .45, *p* = .50 |
| Current alcohol use, n (%) | 53 (77.9) | 32 (65.3) | χ^2^(1, 117) = 2.28, *p* = .13 |
| Current tobacco use, n (%) | 6 (8.8) | 3 (6.1) | *p* = .73^ |
| Years with IBD diagnosis, M (SD) | 10.4 (9.6) | 10.7 (10.4) | *t*(114) = -.17, *p* = .86 |
| HBI categories, n (%) |  |  |  |
| Inactive/remission | 48 (75) |  |  |
| Mild | 9 (14.1) |  |  |
| Moderate | 6 (9.4) |  |  |
| Severe | 1 (1.6) |  |  |
| Partial Mayo score categories, n (%) |  |  |  |
| Inactive/remission |  | 29 (63) |  |
| Mild |  | 12 (26.1) |  |
| Moderate |  | 4 (8.7) |  |
| Severe |  | 1 (1.0) |  |
| Rome IV IBS criteria met, n (%) | 13 (19.1) | 11 (10.1) | χ^2^(1, 117) = .19, *p* = .66 |
| History of surgical resection, n (%) | 26 (38.2) | 3 (6.1) | χ^2^(1, 117) = 15.75, *p* < .001 |
| Current use of aminosalicylate therapy, n (%) | 12 (17.6) | 24 (49.0) | χ^2^(1, 117) = 13.13, *p* < .001 |
| Current use of corticosteroid therapy, n (%) | 5 (7.4) | 9 (18.4) | χ^2^(1, 117) = 3.28, *p* = .07 |
| Current use of immunomodulator therapy, n (%) | 19 (27.9) | 9 (18.4) | χ^2^(1, 117) = 1.43, *p* = .23 |
| Current use of biologic therapy, n (%) | 46 (67.6) | 15 (30.6) | χ^2^(1, 117) = 15.65, *p* < .001 |

*Note.* Statistical significance was met using a *p* value of < 0.003 due to Bonferroni correction to adjust for multiple comparisons. *Maximum likelihood ratio Chi-square test; ^Fisher’s exact test

Supplemental Table 2. Participant psychological functioning and quality of life by type of IBD diagnosis.

|  | Crohn’s Disease | Ulcerative Colitis | Statistical test |
| --- | --- | --- | --- |
| *n* | 68 | 49 |  |
| HADS anxiety score, M (SD) | 7.6 (4.1) | 7.4 (4.5) | *t*(113) = .20, *p* = .84 |
| HADS depression score, M (SD) | 3.9 (3.5) | 3.6 (3.6) | *t*(113) = .32, *p* = .75 |
| PHQ-15 overall score, M (SD) | 7.9 (4.6) | 7.9 (4.7) | *t*(114) = -.05, *p* = .95 |
| SF-36 scores, M (SD) |  |  |  |
| Physical functioning | 84.4 (23.9) | 84.6 (22.5) | *t*(115) = -.04, *p* = .97 |
| Role limits physical health | 75.4 (37.5) | 67.3 (43.4) | *t*(114) = 1.07, *p* = .29 |
| Role limitations emotional problems | 65.7 (40.3) | 73.0 (39.1) | *t*(113) = -.98, *p* = .33 |
| Emotional well-being | 68.7 (20.0) | 70.9 (12.6) | *t*(114) = -.59, *p* = .56 |
| Bodily pain | 71.2 (27.3) | 70.2 (23.6) | *t*(114) = .19, *p* = .85 |
| Social functioning | 75.0 (26.7) | 80.1 (24.7) | *t*(115) = -1.05, *p* = .29 |
| Energy/fatigue | 50.9 (23.3) | 50.6 (24.4) | *t*(114) = .07, *p* = .95 |
| General health problems | 50.4 (20.4) | 51.4 (23.3) | *t*(115) = -.26, *p* = .79 |
| GI Catastrophizing score, M (SD) | 20.2 (10.1) | 20.1 (12.4) | *t*(115) = .05, *p* = .95 |
| IBS Behavioral Responses Questionnaire, M (SD) | 85.3 (26.3) | 83.8 (32.2) | *t*(115) = .29, *p* = .77 |

*Note.* Statistical significance was met using a *p* value of < 0.003 due to Bonferroni correction to adjust for multiple comparisons.
